# Supplementary material for: SomaModules: a pathway enrichment approach tailored to SomaScan data
Source: bioRxiv. 2025 Aug 2:2025.07.30.667673. Preprint. [Version 1] doi: 10.1101/2025.07.30.667673 (PMC12324528; doi:10.1101/2025.07.30.667673)
Supplement: Supplement 1 — Supplementary Table 1: Number of gene sets in each MSigDB collection, derived using different threshold combinations for minimum gene set size and intra-cluster correlation. Supplementary Table 2: Summary of results from WGCNA runs using different sets of parameters. [file media-1.pdf]

ID	Description	MSigDB ID	n = 5			n = 10			n = 20		
			r = 0.3	r = 0.5	r = 0.7	r = 0.3	r = 0.5	r = 0.7	r = 0.3	r = 0.5	r = 0.7
H	Hallmarks	h.all	152	94	58	123	86	58	94	79	55
POS	Positional	c1.all	499	373	268	349	304	253	268	261	243
CGP	Chemical and Genetic Perturbations	c2.cgp	7480	5066	3561	5581	4318	3346	4305	3797	3231
BC	BioCarta	c2.cp.biocarta	405	353	283	293	281	254	234	232	224
K	KEGG	c2.cp.kegg_medicus	621	562	437	431	395	367	366	362	361
KL	KEGG Legacy	c2.cp.kegg_legacy	423	318	222	305	272	210	247	234	199
PID	Pathway Interaction Database	c2.cp.pid	441	365	289	344	323	273	260	252	228
R	Reactome	c2.cp.reactome	2858	2226	1657	2106	1827	1507	1696	1562	1381
WP	WikiPathways	c2.cp.wikipathways	1531	1152	889	1108	973	819	875	824	757
MIR	microRNA Targets	c3.mir.mirdb	6261	4234	2757	4719	3666	2605	3468	3009	2423
MIRL	microRNA Targets Legacy	c3.mir.mir_legacy	580	407	275	464	365	262	341	304	238
TFT	Transcription Factor Targets	c3.tft.gtrd	1273	858	510	1092	789	493	931	730	475
TFTL	Transcription Factor Targets Legacy	c3.tft.tft_legacy	1952	1218	761	1602	1146	743	1192	1031	716
3CA	Curated Cancer Cell Atlas	c4.3ca	361	243	174	223	184	157	162	154	151
CGN	Cancer Gene Neighborhoods	c4.cgn	1038	776	552	798	670	522	599	543	471
CM	Cancer Modules	c4.cm	973	647	491	747	561	455	579	478	424
GOBP	GO Biological Process	c5.go.bp	11224	8200	6103	8351	6970	5672	6880	6214	5402
GOCC	GO Cellular Component	c5.go.cc	1312	954	688	1020	823	649	833	729	615
GOMF	GO Molecular Function	c5.go.mf	1877	1431	1104	1414	1215	1019	1176	1078	968
HPO	Human Phenotype Ontology	c5.hpo	8350	6218	4391	6441	5337	4150	5178	4679	3979
ONC	Oncogenic	c6.all	579	336	213	437	295	204	287	233	194
IMM	Immunologic	c7.immunesigdb	15211	9931	5967	12179	9363	5931	9381	8583	5695
VAX	Vaccine Response	c7.vax	573	406	304	458	365	296	373	343	290
CT	Cell Type	c8.all	2208	1305	898	1694	1177	878	1296	1070	853
**TOTAL**			68182	47673	32852	52279	41705	31123	41021	36781	29573

Supplementary Table 1. Number of gene sets in each MSigDB collection, derived using different threshold combinations for minimum gene set size (n = 5, 10, 20) and minimum intra-cluster correlation (r = 0.3, 0.5, 0.7). Results for the threshold values used in this paper (n=10, r=0.5) are highlighted in grey, which match those reported in Table 1.

power	deepSplit	TOMType	networkType	n_mod	n_soma_mod	n_soma	PC1_var_expl
5	1	none	signed	2	94|44	138	0.329
5	1	none	signed hybrid	2	98|40	138	0.351
5	1	unsigned	signed	2	94|44	138	0.329
5	1	unsigned	signed hybrid	3	84|44|10	138	0.416
5	1	signed	signed	2	94|44	138	0.329
5	1	signed	signed hybrid	3	84|44|10	138	0.416
5	2	none	signed	2	94|44	138	0.329
5	2	none	signed hybrid	3	88|40|10	138	0.43
5	2	unsigned	signed	3	81|44|13	138	0.369
5	2	unsigned	signed hybrid	3	84|44|10	138	0.416
5	2	signed	signed	3	81|44|13	138	0.369
5	2	signed	signed hybrid	3	84|44|10	138	0.416
5	3	none	signed	4	69|43|14|12	138	0.363
5	3	none	signed hybrid	3	88|40|10	138	0.43
5	3	unsigned	signed	3	81|44|13	138	0.369
5	3	unsigned	signed hybrid	3	84|44|10	138	0.416
5	3	signed	signed	3	81|44|13	138	0.369
5	3	signed	signed hybrid	3	84|44|10	138	0.416
5	4	none	signed	6	43|43|17|12|12|11	138	0.337
5	4	none	signed hybrid	3	88|40|10	138	0.43
5	4	unsigned	signed	3	81|44|13	138	0.369
5	4	unsigned	signed hybrid	3	84|44|10	138	0.416
5	4	signed	signed	3	81|44|13	138	0.369
5	4	signed	signed hybrid	3	84|44|10	138	0.416

Supplementary Table 2. Summary of results from WGCNA runs using different sets of parameters: n_mod = number of modules; n_soma_mod = number of SOMAmers in each module; n_soma = number of SOMAmers across all modules; PC1_var_expl = variance explained by Principal Component 1 averaged over all modules. Highlighted in grey are the runs that maximize PC1_var_expl (they all correspond to the same solution).
