# Supplementary material for: SomaModules: a pathway enrichment approach tailored to SomaScan data
Source: bioRxiv. 2025 Aug 2:2025.07.30.667673. Preprint. [Version 1] doi: 10.1101/2025.07.30.667673 (PMC12324528; doi:10.1101/2025.07.30.667673)
Supplement: Supplement 5 — Supplementary Figure 1: Mean correlation density distributions for parent-child SomaModule pairs derived from different MSigDB collections. Supplementary Figure 2: WGCNA grid-search for the optimal soft-thresholding power (beta) for network construction. Supplementary Figure 3: Volcano plots showing differentially abundant SOMAmers from an Alzheimer’s Disease vs control study using 7K SomaScan. Supplementary Figure 4: Paired Student’s t-test significance of enrichment score differences between SomaModules and original MitoCarta pathways for different physical performance outcomes. [file media-5.pdf]

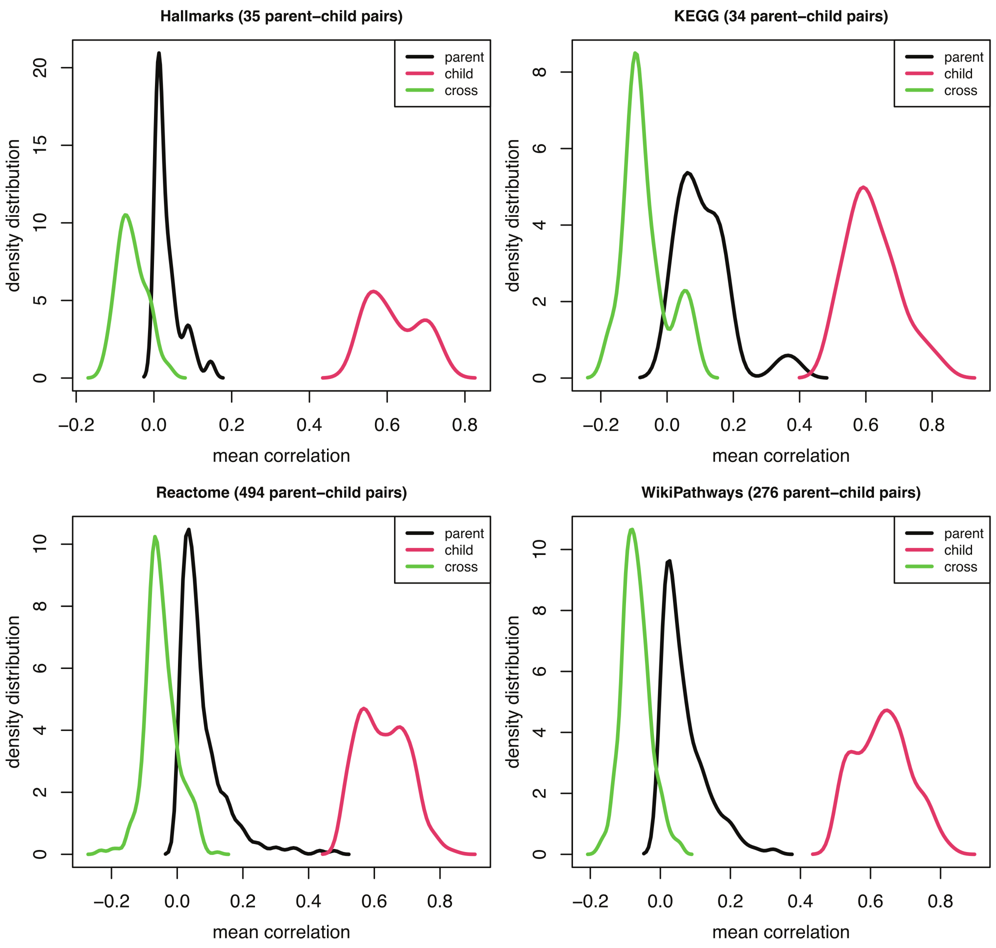

Supplementary Figure 1: Mean correlation density distributions for parent-child SomaModule pairs derived from different MSigDB collections, as indicated.

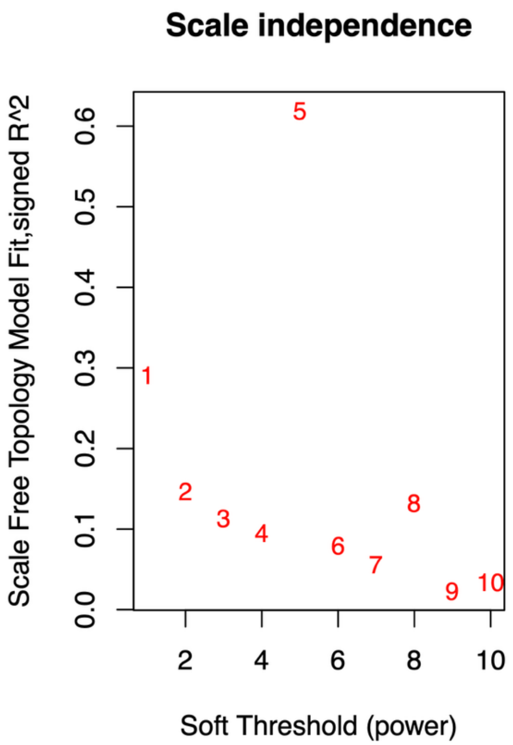

Supplementary Figure 2: WGCNA grid-search for the optimal soft-thresholding power (beta) for network construction.

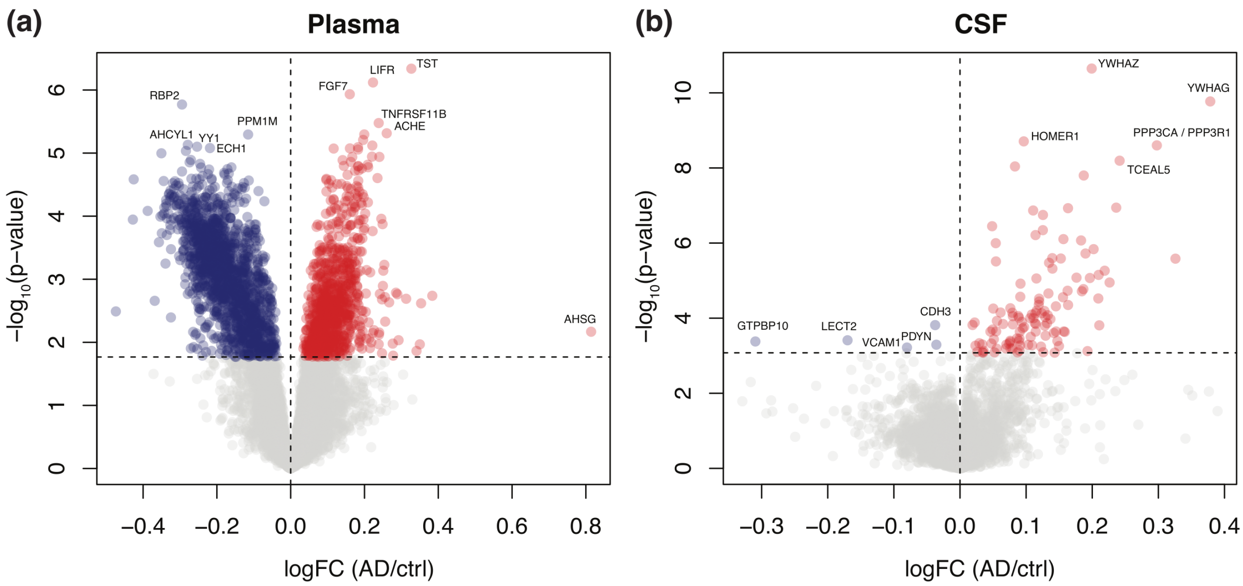

Supplementary Figure 3: Volcano plots showing differentially abundant SOMAmers from an Alzheimer's Disease (AD) vs control study using 7K SomaScan. The horizontal dashed lines show the Benjamini-Hochberg adjusted p-value=0.05. Red symbols show SOMAmers significantly over-abundant in AD relative to controls; blue symbols correspond to SOMAmers significantly over-abundant in controls relative to AD patients. **(a)** Plasma samples (n_{red}=923, n_{blue}=1562). **(b)** Cerebrospinal fluid (CSF) samples (n_{red}=117, n_{blue}=5).

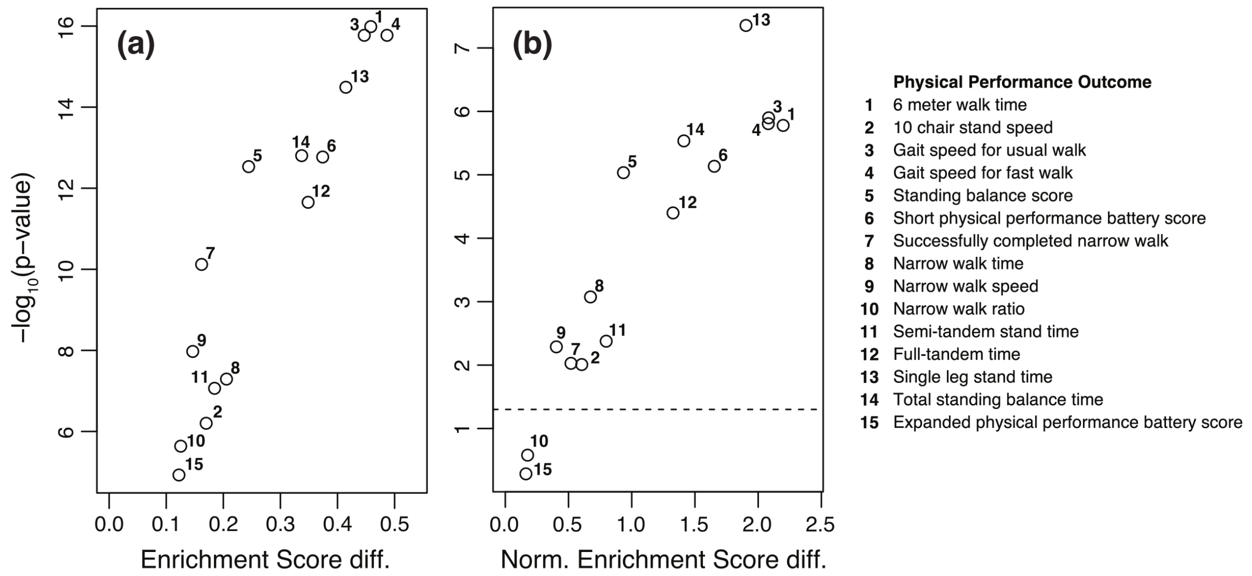

Supplementary Figure 4: Paired Student's t-test significance of enrichment score differences between SomaModules and original MitoCarta pathways for different physical performance outcomes. Panels show differences in **(a)** Enrichment Scores and **(b)** Normalized Enrichment Scores. Physical performance outcomes are listed on the right-hand side. The horizontal dashed line corresponds to p−value = 0.05.
