# Supplementary material for: SomaModules: a pathway enrichment approach tailored to SomaScan data
Source: bioRxiv. 2025 Aug 2:2025.07.30.667673. Preprint. [Version 1] doi: 10.1101/2025.07.30.667673 (PMC12324528; doi:10.1101/2025.07.30.667673)
Supplement: Supplement 6 [file media-6.pdf]

SUPPORTING INFORMATION

SomaModules: a pathway enrichment approach tailored to SomaScan data

Julián Candia*, Giovanna Fantoni, Francheska Delgado-Peraza, Nader Shehadeh, Toshiko Tanaka, Ruin Moaddel, Keenan A. Walker and Luigi Ferrucci

Intramural research Program, National Institute on Aging, National Institutes of Health, Baltimore, MD 21224, USA

* Corresponding author: julian.candia@nih.gov

Table of Contents

Supplementary Table 1: Number of gene sets in each MSigDB collection, derived using different threshold combinations for minimum gene set size and intra-cluster correlation.

Supplementary Table 2: Summary of results from WGCNA runs using different sets of parameters.

Supplementary Figure 1: Mean correlation density distributions for parent-child SomaModule pairs derived from different MSigDB collections.

Supplementary Figure 2: WGCNA grid-search for the optimal soft-thresholding power (beta) for network construction.

Supplementary Figure 3: Volcano plots showing differentially abundant SOMAmers from an Alzheimer's Disease vs control study using 7K SomaScan.

Supplementary Figure 4: Paired Student's t-test significance of enrichment score differences between SomaModules and original MitoCarta pathways for different physical performance outcomes.

Supplementary Data 1: GSEA enrichment scores of AD-specific pathways using 7K SomaScan data from AD vs control plasma samples.

Supplementary Data 2: GSEA enrichment scores of AD-specific pathways using 7K SomaScan data from AD vs control CSF samples.

Supplementary Data 3: GSEA enrichment scores of mitochondrial pathways associated with 15 physical performance metrics using 11K SomaScan data from 2542 BLSA plasma samples.
